# Multi-Channel Vision Transformer for Epileptic Seizure Prediction

**DOI:** 10.3390/biomedicines10071551

**Published:** 2022-06-29

**Authors:** Ramy Hussein, Soojin Lee, Rabab Ward

**Affiliations:** 1 Center for Advanced Functional Neuroimaging, Stanford University, Stanford, CA 94305, USA; 2 Pacific Parkinson’s Research Centre, University of British Columbia, Vancouver, BC V6T 2B5, Canada; soojin.lee@ndcn.ox.ac.uk; 3 Electrical and Computer Engineering, University of British Columbia, Vancouver, BC V6T 1Z4, Canada; rababw@ece.ubc.ca

**Keywords:** EEG, epilepsy, seizure prediction, continuous wavelet transform, vision transformer

## Abstract

Epilepsy is a neurological disorder that causes recurrent seizures and sometimes loss of awareness. Around 30% of epileptic patients continue to have seizures despite taking anti-seizure medication. The ability to predict the future occurrence of seizures would enable the patients to take precautions against probable injuries and administer timely treatment to abort or control impending seizures. In this study, we introduce a Transformer-based approach called Multi-channel Vision Transformer (MViT) for automated and simultaneous learning of the spatio-temporal-spectral features in multi-channel EEG data. Continuous wavelet transform, a simple yet efficient pre-processing approach, is first used for turning the time-series EEG signals into image-like time-frequency representations named Scalograms. Each scalogram is split into a sequence of fixed-size non-overlapping patches, which are then fed as inputs to the MViT for EEG classification. Extensive experiments on three benchmark EEG datasets demonstrate the superiority of the proposed MViT algorithm over the state-of-the-art seizure prediction methods, achieving an average prediction sensitivity of 99.80% for surface EEG and 90.28–91.15% for invasive EEG data.

## 1. Introduction

Epilepsy is a central nervous system disorder that is associated with abnormal electrical activity in the brain [1]. It is characterized by recurrent seizures that strike without warning. Symptoms may include sudden violent convulsions, reduced or suspension of awareness, and sporadically loss of consciousness [2]. Currently, anti-epileptic drugs are the mainstay of epilepsy treatment. Lamentably, around 30% of people with epilepsy continue to have seizures despite treatment [3]. In addition, the other 70% of patients who respond to anti-epileptic medication suffer from several undesirable side effects such as stomach discomfort, tiredness, dizziness, or blurred vision. Epilepsy surgery may be an option when medications fail to control seizures. It is a surgical procedure that removes or disconnects an area of the brain where seizures occur, which helps stop seizures or seize their severity. It may, however, involve serious risks such as visual impairment, memory and language problems, stroke, and paralysis [4]. This motivated researchers to develop seizure prediction solutions [5].

Epileptic seizure prediction holds a great potential for alerting patients of impending seizures so they can take precautions to avoid any probable injuries and administer a fast-acting medication. It also helps pave the way for individualized epilepsy treatment (e.g., tailored therapies with less side-effects), and seizure intervention systems could also be used to abort imminent seizures. Recently, several studies have demonstrated that epileptic seizures could be predicted with reasonable levels of accuracy [6,7], suggesting that epileptic patients can benefit from methods that forecast seizures occurrence far enough in advance. Currently, electroencephalography (EEG) is the most common tool used in seizure detection and prediction studies. The key challenge is to analyze the pre-seizure EEG activities to identify any distinctive pattern(s) that indicate upcoming seizures, which is the main question to address in the proposed study.

Epilepsy researchers have categorized the brain EEG activities of patients with epilepsy into four prime states: preictal (right before seizure), ictal (seizure), postictal (immediately after seizure), and interictal (a seizure-free time period between the postictal and the preictal of consecutive seizures) [8,9]. An ideal seizure prediction algorithm would be able to recognize brain activities during the preictal periods and make correct predictions of future seizure onset (true positive) while minimizing false positive predictions made during the interictal periods (i.e., normal brain activity). To achieve this, several feature engineering and classification approaches have been introduced to differentiate between the preictal and interictal EEG activities. Four main types of EEG features have been used in previous studies: (1) time domain features (e.g., mean, variance, skewness, kurtosis, number of zero-crossing, cross-correlation coefficients) [10], (2) frequency domain features (e.g., spectral entropy, phase locking value, spectral edge frequency, surface cross-frequency coherence) [11], (3) time-frequency domain features (e.g., short-time-Fourier-transform, wavelet sub-bands coefficients, Hilbert/Slant transform) [12], and (4) non-linear features (e.g., Hjorth statistical parameters, Lyapunov characteristic exponent, Hurst exponent, empirical mode decomposition) [13]. Although the above-mentioned hand-crafted features were able to well-characterize different EEG states, they failed to attain clinical applicability due to lack of generalization capacity [14].

In this work, we propose a novel transformer-based algorithm that accurately and robustly classify preictal and interictal EEG activities. The main contributions of our work are as follows: (1) We utilize continuous wavelet transform (CWT), an efficient time-frequency transform, for converting the time-series EEG signals into image-like representations that well maintain both local spectral and temporal EEG information; (2) We introduce a novel multi-channel vision transformer (MViT) model to extract the distinctive temporal-spectral feature representations from different EEG channels simultaneously; and (3) Using both scalp and invasive EEG databases, our approach demonstrates superior seizure prediction performance when compared to the state-of-the-art prediction methods including convolutional and recurrent neural network models.

## 2. Related Work

Over the past decade, researchers have developed seizure-prediction methods utilizing a variety of signal processing and machine learning methods. Before the rise of deep learning, seizure-prediction methods followed a conventional pipeline that consisted of (1) feature engineering to capture the distinguishable EEG features that characterize different classes of EEG activities, and (2) feature stratification using traditional or modern machine learning classification models. The conventional pipeline has been utilized in a seizure advisory system (SAS) that can prognosticate the occurrence of seizures ahead of time using invasive EEG (iEEG) data [15]. The system was implanted in 15 adults with refractory (drug-resistant) epilepsy and achieved a prediction sensitivity in the range of 17–100% with a large subject variability. Another automated seizure prediction solution was developed by Kiral-Kornek et al. [16] based on the invasive EEE data of 10 of the patients who participated in the clinical trial of the SAS. According to a recent review on seizure prediction studies [17], frequency and/or time-frequency domain features are the most prevalent attributes, and support vector machine (SVM)-based algorithms are the most common machine learning classifiers used for EEG classification. For instance, in [18], the authors developed a seizure prediction model based on spectral power features of EEG frequency rhythms. Using a cost-sensitive SVM that can handle the imbalanced class distribution of interictal and preictal samples, the EEG collected from 18 patients in the Freiburg EEG database could be classified with an average sensitivity of 97.5% and a false alarm rate of 0.27/hr. A similar approach (i.e., frequency domain-based EEG features + SVM classifier) has been tested on other datasets [19,20,21], with performance ranging between 90 and 92% prediction sensitivity. Williamson et al. [22] proposed to utilize multivariate EEG features instead of the popular univariate features such as the power spectral density to capture patterns involving multiple EEG channels. An SVM classifier was trained on the multivariate EEG features and resulted in Area under the ROC Curve (AUC) score of 0.936–0.972 when tested on 19 patients in the Freiburg EEG database.

The advent of deep learning has transformed and advanced the field of epileptic seizure prediction. In particular, end-to-end automated learning taking advantage of deep neural networks has enabled bypassing the laborious feature extraction and selection processes while successfully solving the challenging task of predicting seizures solely from neural signals. Several deep learning models that use time-frequency representations (e.g., spectrograms) of the EEG data as the input have been proposed. In [23], a convolutional neural network (CNN) consisting of six convolutional layers and two fully connected layers was proposed to distinguish between the interictal and preictal EEG activities of two independent scalp EEG datasets. Trained on 3D wavelet tensors computed from the Wavelet transformation of raw scalp EEG data, the CNN achieved a prediction sensitivity of 87.8% and a false prediction rate of 0.142/hr. A similar line of work can be found in [24], where raw EEG signals were transformed into time-frequency features using short-time Fourier transform (STFT), and the produced image-like 2D features were used as inputs to a CNN consisting of three convolution blocks and two fully connected layers. The proposed model achieved a sensitivity of 81.4%, 81.2%, and 75% when tested on the Freiburg Hospital dataset, CBH-MIT dataset, and the American Epilepsy Society (AES) Seizure Prediction dataset, respectively. Wang et al. [25] trained a CNN model (3 convolutional layers followed by 3 fully connected layers) on channel-frequency feature maps derived by the directed transfer function (DFT) and achieved a sensitivity of 90.8% and false prediction rate of 0.08/h on the Freiburg EEG dataset.

Several studies have proposed a different line of CNN architecture. Instead of the 2D-CNN models described above, Ozcan et al. [26] adopted a 3D-CNN model trained on the time and frequency features of EEG data and achieved a sensitivity of 85.7% when tested on the CHB–MIT dataset. Liu et al. [27] introduced a multi-view CNN architecture to simultaneously utilize information from the frequency and time domains. Specifically, fast Fourier transform (FFT) was applied to the raw EEG signals to obtain a representation in the frequency domain, and time domain features were obtained using approaches such as autoregression coefficient, correlation, and signal entropy. The two sets of domain-based features are separately processed through a series of convolutional layers, and then a fully connected layer was used to combine the two different views into a more representative single shared view. The proposed model was used to predict the occurrence of epileptic seizures and resulted in an average AUC score of 0.837 on the AES invasive EEG dataset and AUC scores of 0.82 and 0.89 on two subjects of the CHB–MIT surface EEG dataset. In [28], a novel CNN architecture with multi-scale temporal convolution was introduced to tackle the common domain-shift problem (between training and testing samples) in the seizure prediction field. The proposed model encodes preictal features in different time spans to constrain the consistency of features between training and testing samples. The model was found to learn effective features against signal pattern shifting and improve seizure prediction performance by around 9% when tested on two public datasets of Freiburg and AES Kaggle competition.

Hussein et al. [29] proposed a semi-dilated convolutional network (SDCN) architecture capable of effectively expanding the receptive field of convolution filters along the long dimension (time) while preserving the high resolution along the short dimension (frequency) when applied to EEG scalogram images. Their proposed semi-dilated CNN achieved a high seizure-prediction sensitivity of 98.8% and 88.5–89.52% for a public scalp EEG and two invasive EEE datasets, respectively. More recently, novel seizure-prediction algorithms utilizing graph convolutional networks (GCNs) were introduced. GCN-based seizure-prediction methods consider EEG channels as nodes and their relationships as edges to build a topological graph, which helps leverage graphical structures of multiple channels. Lian et al. [30] proposed a joint graph structure and representation learning network to capture both the global and local contextual information of EEG signals. Using the Freiburg dataset, this network demonstrated promising results achieving superior prediction accuracy and sensitivity compared to other classifiers such as SVM, XGBoost, and 2D-CNN.

## 3. Materials and Methods

### 3.1. Datasets

We evaluate the representation learning capabilities of the proposed MViT on both scalp and invasive EEG data recorded from pediatric and adult humans as well as dogs. The following public datasets are used for benchmarking the proposed MViT and baseline seizure-prediction methods:

**CHB–MIT Scalp EEG Dataset** [31]—This database, acquired at the Children’s Hospital Boston (CHB), contains EEG recordings from pediatric participants with drug-resistant epilepsy. The patients were monitored for several days after the withdrawal of anti-epileptic medication to evaluate their condition for surgical intervention. EEG recordings were collected from 22 patients (5 males, age: 3–22; 17 females, age: 1.5–19) at 256 Hz sampling frequency and 16-bit resolution. Around 9–42 continuous 1-h EEG measurements were recorded for each patient. For most of the patients, the international 10–20 system for EEG electrode placement was adopted and 23 electrodes were used to record the multi-channel EEG data. More electrodes were used for a few patients, where 24 and 26 electrodes were used to record more detailed EEG data. A total of 198 seizure events were recorded in the CHB–MIT dataset, and the onset and termination of the seizures were labeled and provided together with the EEG measurements.

**Kaggle/American Epilepsy Society (AES) Invasive EEG Dataset** [32]—This EEG dataset was collected from two adult human and five canine subjects. The EEG signals were recorded at a sampling frequency of 400 Hz using an ambulatory 16-electrode system. The five dogs produced high-quality EEG recordings with sufficient number of seizures, spanning 7–12 months. The two human subjects were diagnosed with drug-resistant epilepsy. The first patient (female, 70 years old) underwent iEEG monitoring for 71.3 h with five seizures recorded. The second patient (female, 48 years old) had intractable epilepsy and underwent 158.5 h of iEEG monitoring. All iEEG data were organized into 10-min EEG clips labeled “preictal” for pre-seizure data and “interictal” for inter-seizure (between seizures) data. Preictal EEG data clips are provided covering one hour before seizure with a five minute offset (i.e., the five minutes before seizure onset). Similarly, the 10-min interictal EEG data clips were chosen randomly from the full EEG recordings, with the restriction that interictal clips be more than 4 h before or after any seizure, to avoid contamination with either preictal or postictal data.

**Figure 1:**
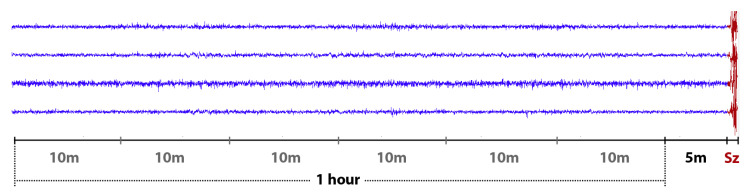
Examples of one-hour preictal (pre-seizure) EEG signals with a 5-min offset before seizures; **Sz** denotes the seizure onset. For convenience, only four channels are plotted.

**Kaggle/Melbourne University Invasive EEG Dataset** [33]—This invasive EEG dataset was recorded from three adult patients suffering from drug-resistant focal epilepsy using the NeuroVista Seizure Advisory System (described in [15]). The first patient (female, 22 years old) was diagnosed with focal epilepsy at age 16. She was treated with several anti-epileptic medications and had epilepsy surgery before the clinical trial. The second patient (female, 51 years old) was also diagnosed with focal epilepsy at age 10. She was receiving a Carbamazepine drug at the time of the clinical trial. The third patient (female, 50 years old) was diagnosed with frontal lobe epilepsy at age 15 and underwent epilepsy surgery before the clinical trial. Sixteen electrodes were implanted on the surface of the brain, directed to the brain regions with presumed seizure focus, and connected to a wireless module embedded in the subclavicular area. Data were sampled at 400 Hz, digitized with 16-bit resolution, transmitted to an external hand-held advisory device, and stored in a removable flash drive. As in the American Epilepsy Society iEEG dataset, both preictal and interictal iEEG data were split into 10-min clips. Preictal data were also provided covering one hour before seizure with a five-minute offset segment. Figure 1 shows examples of the 1-h preictal data recorded from four EEG channels. Interictal data clips were also segmented from 60-min long recordings that started at an arbitrarily time with a minimum gap of 4 h before or after any seizure.

### 3.2. Methodology

Our proposed multi-channel vision transformer is inspired by the success of the vision transformer of Dosovitskiy et al. [34], which showed promising results for several image recognition tasks. We propose a variant of the vision transformer with multiple-path architecture to extract multi-channel EEG features for better seizure prediction. The proposed architecture comprises different branches that concurrently operate at different EEG channels to learn and integrate the distinctive tempo-spectral features needed for reliable EEG stratification.

#### 3.2.1. EEG Pre-Processing

The proposed EEG pre-processing strategy comprises two major procedures: (1) EEG segmentation, in which the time-series EEG clips are split into shorter non-overlapping EEG segments; and (2) encoding the resulting EEG segments into image-like representations using continuous wavelet transform. The output representations are then fed into the proposed MViT model for EEG feature learning and classification.

**EEG Segmentation:** Both surface and intracranial EEG signals are characterized as non-stationary data, i.e., their statistical features change over time [35]. The main rationale behind EEG segmentation is to split the non-stationary EEG signal into shorter pseudo-stationary chunks with comparable statistical properties [36]. Additionally, EEG segmentation can significantly increase the number of labeled data samples needed for improving the performance of vision transformers. In this work, each 10-min EEG clip is split into 60 non-overlapping segments; each is 10 s long. This results in a 60-fold increase in the total number of both interictal and preictal EEG samples.

**Figure 2:**
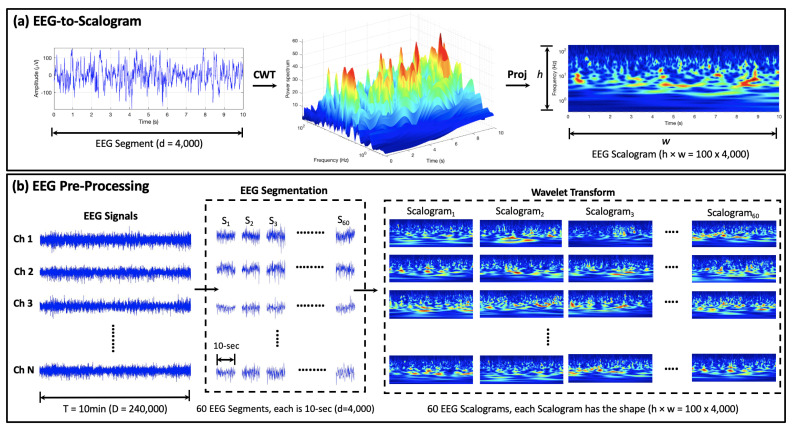
Schematic pipeline of the proposed EEG pre-processing strategy for seizure prediction: (**a**) EEG-to-scalogram conversion procedure: continuous wavelet transform (CWT) is adopted to generate the EEG power spectrum from the time-series EEG data; and 3D-to-2D projection (Proj) is used to produce the 2D time-frequency representations of EEG named “scalogram”. (**b**) EEG pre-processing approach: $S_{1}$, $S_{2}$, ⋯, $S_{60}$ correspond to the 1st, 2nd, and 60th 10-s segments of each 10-min EEG clip ($f_{S}$ = 400 Hz); *N* is the total number of EEG channels (*N* = 23 for scalp EEG; *N* = 16 for invasive EEG); *d* is the number of data-points in each EEG segment (*d* = 10-s ×$f_{S}$ = 4000); and *h* and *w* are the height and width of the EEG scalogram images (h×w = 100 × 4000).

**Mapping EEG Segments into Images:** The automatic detection of different EEG brain statuses is clinically useful for both seizure-detection and -prediction tasks. Although several approaches exist based on the hand-crafted temporal or spectral EEG features, they result in a high number of false positives. Wavelet transform, a time-frequency analysis tool, can effectively attain both temporal and spectral characteristics in a single image-like representation called a “scalogram”. After EEG segmentation, continuous wavelet transform is applied to the 10-s EEG segments to generate the two-dimensional EEG scalogram images, which are then used as inputs to the MViT approach. Figure 2a shows the EEG-to-scalogram conversion procedure, where the left panel shows an example of a 10-s preictal EEG segment (with $f_{S}$ = 400 Hz) and the middle and right panels show the corresponding EEG power spectrum in 3D and 2D domains, respectively. It is worth mentioning that the scalogram of the interictal EEG data (omitted for lack of space) reveals a lower power spectrum in the same time-frequency scales. This contrast in the characteristics of the scalogram images underlying interictal or preictal activities can be exploited to build an efficient seizure prediction system.

The initial data shape is *M* × *N* × *D*, where *M* is the total number of EEG samples, *N* is the number of EEG channels (23 for scalp EEG; 16 for invasive EEG), and *D* is the length of the 10-min EEG clip (153,600 for surface EEG; 240,000 for invasive EEG). After data segmentation and reshaping, the resulting shape of the EEG data is 60M × *N* × *d*, where 60 is the number of the 10-s EEG segments in each 10-min EEG clip, and *d* is the length of the 10-s EEG segment (2560 for surface EEG; 4000 for invasive EEG). Since CWT is applied to each EEG channel individually, the resulting shape of the data is thus 60M × *N* × *h* × *w*, where *h* and *w* are the height and width of the EEG scalogram images. Figure 2b depicts all the transformations applied to the raw EEG data before feeding it to the MViT approach for representation learning and classification.

#### 3.2.2. MViT for EEG Representation Learning

Transformer is a new type of neural network architecture that was originally introduced for natural language processing (NLP) tasks [37], where multi-layer perceptron (MLP) layers are used on top of multi-head attention mechanisms to capture the long-term dependencies in sequential data. Recently, vision transformer (ViT) showed great potential in several computer vision tasks, including image classification [34] and segmentation [38]. Motivated by this, we propose a ViT variant called multi-channel vision transformer (MViT) that operates at different EEG channels simultaneously. More specifically, we introduce a ViT architecture with multiple branches where each branch processes a different EEG scalogram image, and then the information from the different branches is aggregated and used for EEG classification.

**Figure 3:**
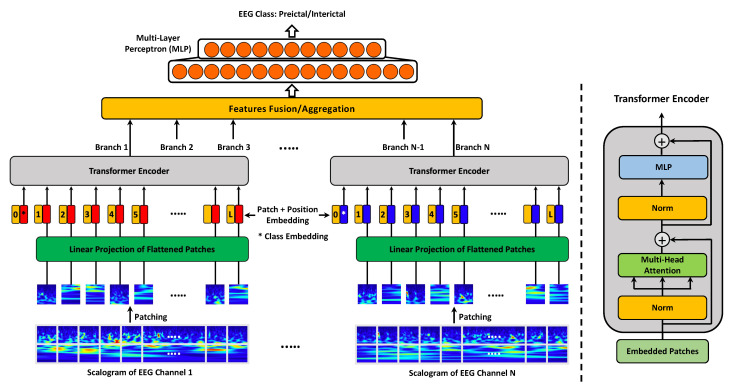
Framework of MViT for multi-channel EEG feature learning. It consists of a stack of *N* transformer encoders; each encoder processes image tokens from an individual EEG channel. The output feature representations are then concatenated and fed as an input to MLP for EEG classification.

Figure 3 illustrates the network architecture of our MViT approach. The model is primarily composed of *N* transformer encoders, where each encoder takes one of the *N* EEG scalograms as an input. Each 2D scalogram image $x\in R^{H\times W}$ is first split into fixed-size non-overlapping 2D patches $x_{p}\in R^{L\times P^{2}}$, where (H×W) is the shape of the EEG scalogram image; (P×P) is the shape of the resulting patches; and $L=HW/P^{2}$ is the number of patches, which also represents the length of the input sequence for each of the MViT branches. The resulting patches are then flattened and mapped into lower-dimensional representations called “patch embeddings” using linear projection. The size of the patch embeddings is set to *D*, which also is the size of the latent vector used by the transformer through all of its layers.

Since position information is crucial for computer vision tasks, position embeddings are also added to the patch embeddings and the resulting sequence of embedding vectors serves as an input to the transformer encoder. The standard transformer encoder presented in [37] is used for EEG scalogram encoding and representation learning. The multi-head self attention (MSA) and multi-layer perceptron (MLP) are the main blocks in the transformer encoder as they help attain both local and global dependencies in the input images. Layer normalization (LN) is applied before each block to improve the accuracy and training time, and the residual connections are also included after each block as they allow the components to flow through the network directly without passing through non-linear activations. Lastly, the output feature representations of the different transformer encoders are aggregated and used as an input to MLP for preictal/interictal EEG classification.

#### 3.2.3. Performance Evaluation

Several performance metrics including accuracy (ACC), sensitivity (SENS), specificity (SPEC), false-positive rate (FPR), and area under the ROC curve (AUC) are used to evaluate the performance of the proposed MViT approach for epileptic seizure prediction.

## 4. Results and Discussion

In this section, we evaluate the seizure prediction performance of the proposed MViT approach and compare it to the concurrent and previous works when examined on the same benchmark surface and invasive EEG databases.

### 4.1. MViT Prediction Performance on Surface Pediatric EEG

In this section, the seizure prediction performance of our proposed MViT algorithm is compared to the classical machine learning and recent deep learning methods on the CHB–MIT surface EEG dataset. Table 1 reports the performance metrics achieved by the proposed, concurrent, and previous seizure-prediction methods. In [39,40,41], the SVM classifier was used together with a set of domain-based hand-picked EEG features, namely, spectral power, phase looking value, and spectral moments, yielding a seizure-prediction sensitivity between 82.4% and 98.7%. The methods introduced in [39,40,41], however, rely on domain-based features that are usually unreliable and prone to domain shift. Thus, the discriminative power of such prediction systems is negatively affected especially when tested on unseen data. Our MViT algorithm, on the contrary, extracts the distinguishable EEG features in an automated manner, achieving higher seizure-prediction sensitivity of 99.8%, as shown in Table 1.

We also compare our MViT approach with other concurrent and recently-developed deep learning methods that either use CNN [23,24,43,47,49,54] or long short-term memory (LSTM) [42,44] for epileptic seizure prediction. In [23], the raw EEG signals were converted to 3D wavelet tensors (time × scales × channels) and fed into a CNN model, which achieved a prediction sensitivity of 86.6% and a FPR of 0.147/h. In [24], the raw EEG data were converted into image-like 2D representations using STFT and then fed into a three-block CNN architecture. The results showed an average seizure-prediction sensitivity of 81.2% and a FPR of 0.16/h. In [43], the spectral power of EEG rhythms was used as inputs to a three-layer CNN model for automated EEG feature learning and classification. The results showed that both seizure-prediction sensitivity and FPR were considerably improved to 92.0% and 0.12, respectively. More recently, several studies have demonstrated that CNN-based models can be also effectively applied to raw EEG signals and achieve comparable prediction performance with a sensitivity of 92.0–98.8% [47,49,54].

Recurrent neural networks (RNNs) were also used for predicting epileptic seizures based on EEG signals. For instance, Tsiouris et al. [42] employed a LSTM network—a common type of RNN—to forecast the occurrence of seizure onsets in the CHB–MIT surface EEG recordings. Although the results showed high levels of seizure-prediction sensitivity and specificity (≈99%), the LSTM-based solution cannot be generalized to new patients as the network was trained and tested in a subject-specific manner. Additionally, two patient-specific seizure prediction solutions that use LSTM were also presented in [44]. They achieved high seizure prediction performance of 99.7% sensitivity and 0.004/h FPR. However, their LSTM and deep convolutional autoencoder (DCAE) networks were trained and tested on individual patients to forecast the patient-specific seizure onsets. Our MViT approach, on the other hand, is trained and tested on the CHB–MIT dataset using leave-one-subject-out cross-validation, which demonstrates the ability of our approach to generalize and maintain robust seizure prediction performance on unseen EEG data recorded from new patients. The results reveal that the proposed MViT feature learning approach, together with wavelet transform, yields superior seizure prediction performance, achieving an average specificity, accuracy, and FPR of 99.7%, 99.8%, and 0.004, respectively.

### 4.2. MViT Prediction Performance on Invasive Human and Canine EEG

In this section, we also evaluate the seizure prediction performance of our MViT approach on the invasive human and canine EEG data of the Kaggle/AES dataset [32]. The model is trained and tested on humans and dogs individually. The prediction performance of the MViT is compared to the top five Kaggle algorithms [32] and other recent machine and deep learning methods [24,27,28,29,56,57,58,59,60,61]. As shown in Table 2, the top five algorithms of the Kaggle/AES dataset achieved AUC scores between 0.825 and 0.903 when tested on the Kaggle/AES public test set and 0.793–0.840 when tested on the Kaggle/AES private test set. The highest prediction scores achieved by the top Kaggle winning teams were based on both frequency domain features such as spectral entropy and spectral power, as well as on non-linear representations such as fractal dimensions and Hurst exponents. These features were extracted from the frequency rhythms of the EEG signals and fed into an efficient classification model to differentiate between the interictal and preictal EEG activities. Among all classifiers, the SVM, Random Forest, and Lasso regularization of generalized linear models (LassoGLM) were found to achieve the highest AUC scores. Neural networks, however, were found to achieve inferior results of 0.825/0.793 AUC when used with a combination of spectral features, temporal features, and principal component analysis (PCA). Our MViT algorithm, on the other hand, exhibits remarkable improvements on both human and canine EEG data, achieving average AUC scores of 0.940 and 0.885 on the Kaggle/AES public and private test sets, respectively.

Table 2 also compares other machine learning methods applied to the American Epilepsy Society dataset. In [24], Truong et al. used STFT for EEG pre-processing and then adopted a generic CNN architecture for EEG stratification. They first segmented the 10-min invasive EEG clips into 5-s non-overlapping chunks, then utilized STFT for transforming these time-series chunks into spectrogram images, and finally supplied the EEG spectrograms to a CNN for the automatic learning and classification of interictal and preictal EEG activities. This method helped achieve an average seizure-prediction sensitivity of 75% on both human and canine EEG data. Our seizure-prediction algorithm uses a more adequate pre-processing approach that relies on CWT for data transformation but with a more efficient architecture that learns temporal-spectral feature representations from different EEG channels simultaneously. As shown in Table 2, our MViT algorithm produces a notable seizure-prediction sensitivity of 90.28%.

We also demonstrate a benchmark of the recently-developed deep learning methods [27,56,57,58] and our seizure prediction method. In [56], a simple CNN architecture that adopts one-dimensional convolutions was applied to the multi-channel invasive EEG data, resulting in an average AUC score of 0.843 on the public test set. Moreover, Ma et al. implemented and examined the LSTM—a recurrent neural network architecture—for the seizure prediction problem under study [57]. Unlike traditional approaches that use time-series data as an input to the LSTM, Ma et al. provided the statistical features extracted from EEG rhythms as inputs to the proposed LSTM-based seizure prediction approach. This resulted in a better AUC score of 0.894 when tested on the public test set. In [58], the spectral power features of invasive EEG signals were also used as inputs to a CNN model that achieved an average AUC score of 0.780 on the public test set and 0.760 on the private test set. In [27], a multi-view CNN architecture was introduced to capture multi-scale EEG features, and an average AUC score of 0.837 and 0.842 was achieved for public and private test sets, respectively.

The combination of EEG spectrogram (generated by STFT) and CNN was also used in [59], yielding a limited seizure-prediction sensitivity of 82% and an AUC score of 0.746 on the public test set. Improved seizure-prediction results (AUC scores of 0.928 and 0.856) were reported in [29], where the authors applied a novel semi-dilated convolutional network to the scalograms of the invasive EEG data. Compared with the traditional convolutions, the semi-dilated CNN was found to boost the seizure prediction accuracy by exploiting the wide temporal-level and fine-grained spectral-level information needed to distinguish between preictal and interictal EEG data.

In [60,61], customized seizure-prediction results were reported. Usman et al. [60], for instance, introduced a patient-dependent seizure prediction solution that uses a set of temporal and spectral hand-crafted EEG features as an input to an ensemble classifier of SVM, CNN, and LSTM. On the contrary, Zhao et al. [61] used the raw invasive EEG data as inputs to a CNN model whose architecture was automatically determined via neural architecture search instead of being manually designed. The models proposed in [60,61] were trained and tested on individual subjects and achieved promising patient-specific seizure-prediction results (sensitivity of 91.77–94.20%). Our proposed MViT model has been found to outperform the existing CNN- and LSTM-based methods by a significant margin, producing an average AUC score of 0.940 on the public test set. More importantly, our seizure predictor achieves the highest AUC score of 0.885 on the unseen data of the private test set, proving that it can accommodate the variations in EEG data across different subjects and also over time for the same subject. This makes our MViT model an excellent candidate for clinical and real-life settings.

### 4.3. MViT Prediction Performance on Invasive Human EEG

In this section, we test our seizure-prediction algorithm on the invasive EEG data of the Kaggle/Melbourne University seizure prediction dataset [33]. The data was collected from three adult human subjects, all females, who had epilepsy surgery before the clinical trial. We compare the prediction performance of our MViT approach with the top winning teams of the Kaggle competition [33] as well as baseline machine learning methods [15,16,29,61,62,63,64]. In [15], Cook and his team successfully implanted the first-in-man seizure advisory system in several patients with drug-resistant epilepsy. After the system implantation, a seizure forecasting method was introduced to identify time intervals of the low, medium, and high occurrence probability of impending seizures. The initial seizure-prediction results were satisfactory for most of the subjects, proving that seizure prediction using EEG is possible. The average seizure-prediction sensitivity for all patients was 61.20%, while the three patients under study had the least seizure-prediction sensitivities with an average of 33.67%. The major cause of this performance degradation for these particular three patients was the data drift observed in the temporal EEG features used for prediction [15]. Improving the prediction performance for these three patients is important to ensure that seizure prediction is feasible for different patients, including those whose EEG characteristics vary over time.

In [62], Karoly et al. developed a circadian seizure forecasting approach to identify pre-seizure brain activities. They proposed to use the spike rate in preictal EEG recordings as a biomarker that indicates whether the brain is approaching an eminent seizure. This biomarker, however, was proven to be unreliable and cannot be generalized to all patients. The spike rate was found to increase before seizures for nine patients and decrease before seizures for the remaining six patients. They used logistic regression to evaluate the effectiveness of their temporal feature engineering approach, which showed an average prediction sensitivity of 62.10% for all the 15 patients and 52.67% for the three patients under study. In [16], Kiral-Kornek et al. proposed to use deep learning for developing patient-specific seizure warning systems that could be fine-tuned to meet patients’ needs. Their work manifested a significant improvement in the seizure prediction performance for almost all patients. An average prediction sensitivity of 69.00% was achieved for the 15 patients, while the three patients whose data are studied in this work had an average prediction sensitivity of 77.36%. In our study, a multi-channel vision transformer approach was neatly developed to thoroughly search the hidden pre-seizure patterns and thus improve the seizure prediction for those three patients. Table 3 reports the seizure-prediction results achieved by the proposed MViT approach, along with the concurrent and previous seizure prediction studies. It should be noted that our approach achieves a superior seizure-prediction sensitivity of 91.15% for the three patients under study.

In addition, we compare our prediction results to those of the winning solutions of the Kaggle/Melbourne University seizure prediction competition. The AUC score was the performance metric used for ranking the submitted solutions. The winning team employed eleven different machine learning classifiers with more than 3000 hand-engineered EEG features and achieved an average AUC score of 0.854 on the public test set and 0.791 on the private test set (see Table 3) [33]. However, it is impractical to deploy such computationally intensive and manually extracted EEG features in real-time applications. In general, the top five Kaggle solutions used a variety of hand-crafted features attained in the time domain, frequency domain, or time-frequency domain. The SVM, adaptive boosting, tree ensemble, and random forest classifiers were used to assess the usefulness of the extracted domain-based EEG features. The results of the top five solutions showed an average AUC score of 0.783–0.854 on the public test set and 0.746–0.807 on the private test set [33]. The authors of [63] also studied the possibility of combining the preictal probabilities produced by the top eight competition solutions. This ensemble approach, however, failed to improve the prediction performance, achieving an average AUC score of 0.815 on the public test set.

**Table 3:** Benchmarking of the previous seizure-prediction methods and our MViT approach: Melbourne University AES/MathWorks/NIH Seizure Prediction dataset.

Authors/ Team	Year	EEG Features	Classifier	SENS (%)	AUC Score Public/Private
Cook et al. [15] ^☆^	2013	Signal energy	Decision tree, kNN	33.67	-
Karoly et al. [62] ^☆^	2017	Signal energy, circadian profile	Logistic regression	52.67	-
Kiral-Kornek et al. [16] ^☆^	2018	EEG Spectrogram, circadian profile	CNN	77.36	-
Not-so-random	2018	Hurst exponent, spectral power,	Extreme gradient	-	0.853/0.807
-anymore [33]		distribution attributes, fractal dimensions,	boosting,		
		AR error, and cross-frequency coherence	kNN, SVM		
Arete	2018	Correlation, entropy, zero-crossings,	Extremely	-	0.783/0.799
Associates [33]		distribution statistics, and spectral power	randomized trees		
GarethJones [33]	2018	Distribution statistics, spectral power,	SVM	-	0.815/0.797
		signal RMS, correlation, and spectral edge	tree ensemble		
QingnanTang [33]	2018	Spectral power, spectral entropy	Gradient boosting,	-	0.854/0.791
		correlation, and spectral edge power	SVM		
Nullset [33]	2018	Hjorth parameters, spectral power,	Random Forest,	-	0.844/0.746
		spectral edge, spectral entropy,	adaptive boosting,		
		Shannon entropy, and fractal dimensions	and gradient boosting		
Reuben et al. [63]	2019	Preictal probabilities from	MLP	-	0.815/-
		the top 8 teams in [33]			
Varnosfaderani et al. [64]	2021	Temporal features, statistical moments,	LSTM	86.80	0.920/-
		and spectral power			
Hussein et al. [29]	2021	EEG Scalogram	SDCN	89.52	0.883/-
Zhao et al. [61]	2022	Raw EEG	CNN	85.19–86.27	0.914–0.933/-
Proposed Method	2022	EEG Scalogram	MViT	91.15	0.924/-

^☆^ Patients 1, 2, and 3 in the Melbourne University Kaggle competition dataset are the same as Patients 3, 9, and 11 in [15,16,62].

Two recent studies [29,61] achieved improved AUC scores (Table 3) by training CNNs on time-frequency features [29] and raw EEG signals [61], respectively. The promising results of the two studies were obtained using considerably different forms of the inputs, which demonstrates the versatility of CNNs in the seizure prediction task. However, the results from [61] are based on the model trained and tested on individual subjects, and the generalizability of CNNs trained on raw EEG signals needs to be validated in future studies. Varnosfaderani et al. [64] reported a higher AUC score of 0.920 using a two-layer LSTM network. The authors first extracted hand-crafted features including temporal features (e.g., mean, variance, and peak-to-peak values) and spectral features (e.g., spectral power in eight canonical EEG frequency bands) from the EEG signals and used them as inputs to the LSTM network. Our proposed MViT algorithm, on the other hand, achieves a superior AUC score of 0.924 while relaxing the need for manually extracting domain-based features. The MViT algorithm is also much faster in obtaining the results on unseen data and is thus more suitable for use in ambulatory and clinical applications.

## 5. Clinical Significance and Limitations

**Clinical significance:** This study’s findings reveal how vision transformers can be effectively adopted for simultaneous feature learning of multi-channel EEG data. The findings would be of major importance in evaluating how distinctive EEG representations extracted by a vision transformer can markedly improve the seizure prediction performance using both surface and invasive EEG data. The proposed multi-channel vision transformer (MViT) algorithm achieved a high prediction sensitivity of 90.28–99.80% across three independent public datasets, demonstrating its potential clinical application as a remote EEG-based seizure warning system. The proposed MViT approach can accurately and rapidly forecast future seizure onsets, providing patients with opportunities to take fast-acting medications and safety measures during the periods of great seizure susceptibility. Closed-loop seizure intervention systems could also be adopted to abort imminent seizures for patients with drug-resistant epilepsy.

**Limitations:** Despite promising seizure-prediction results for vision transformer-based models, there exist several challenges related to their applicability in clinical settings. As highlighted in [65], large-scale vision transformers can require intensive power and computational resources, limiting their deployment on resource-constrained devices such as brain-computer interface and seizure warning systems. It is also quite challenging to interpret vision transformers’ decisions [66], e.g., by visualizing the image regions with the greatest impact on the EEG classification performance. Vision transformers, however, were proven to be more robust than convolutional and recurrent neural networks against texture changes and data contamination, making them more generalizable and reliable in real-life settings [67]. Current works focus on reducing the high computational cost of vision transformers (caused by the self-attention mechanisms [37]) by developing a computationally-efficient self-attention mechanism that can accommodate high-resolution images on resource-constrained systems without compromising accuracy.

## 6. Conclusions

In this study, we proposed a multi-channel vision transformer (MViT) algorithm for the accurate prediction of epileptic seizures. The EEG signals were first divided into shorter non-overlapping chunks of 10-s duration each. Continuous wavelet transform was then adopted to convert the resulting EEG chunks into image-like representations named “scalograms”. The scalogram images were then split into fixed-size non-overlapping patches, which were used as inputs to the MViT algorithm to automatically learn the distinctive EEG features needed for subtle seizure prediction. The proposed MViT architecture comprises multiple branches where each branch operates at a typical EEG channel, allowing learning temporal-spectral features from the different EEG channels simultaneously. With extensive experiments, we demonstrate that the proposed MViT model outperforms several concurrent and previous works on seizure prediction including advanced convolutional and recurrent neural network models.

## Figures and Tables

**Table 1:** Benchmarking of the previous seizure-prediction methods and our MViT approach: CHB–MIT EEG dataset.

Authors	Year	EEG Features	Classifier	SENS (%)	SPEC (%)	ACC (%)	FPR (/h)
Zhang and Parhi [39]	2016	Spectral power	SVM	98.7	-	-	0.04
Cho et al. [40]	2016	Phase locking value	SVM	82.4	82.8	-	-
Usman et al. [41]	2017	Statistical and spectral moments	SVM	92.2	-	-	-
Khan et al. [23]	2018	Wavelet coefficients	CNN	86.6	-	-	0.147
Truong et al. [24]	2018	EEG Spectrogram	CNN	81.2	-	-	0.16
Tsiouris et al. [42]	2018	Spectral power, statistical moments	LSTM	99.3–99.8	99.3–99.9	-	0.02–0.11
Ozcan et al. [26]	2018	Spectral power, statistical moments	3D CNN	85.7	-	-	0.096
Zhang et al. [43]	2019	Common spatial patterns	CNN	92.0	-	90.0	0.12
Daoud et al. [44]	2019	Multi-channel time series	LSTM	99.7	99.6	99.7	0.004
Usman et al. [45]	2020	EEG Spectrogram + CNN features	SVM	92.7	90.8	-	-
Büyükçakır et al. [46]	2020	Statiscal moments, spectral power	MLP	89.8	-	-	0.081
Xu et al. [47]	2020	Raw EEG	CNN	98.8	-	-	0.074
Dissanayake et al. [48]	2021	Mel-frequency cepstral coefficients	Siamese NN	92.5	89.9	91.5	-
Hussein et al. [29]	2021	Scalogram	SDCN	98.9	-	-	-
Jana et al. [49]	2021	Raw EEG	CNN	92.0	86.4	-	0.136
Li et al. [50]	2021	Spectral-temporal features	GCN	95.5	-	-	0.109
Usman et al. [51]	2021	EEG Spectrogram	LSTM	93.0	92.5	-	-
Yang et al. [52]	2021	EEG Spectrogram	Residual network	89.3	93.0	92.1	-
Dissanayake et al. [53]	2022	Mel frequency cepstral coefficients	GNN	94.5	94.2	95.4	-
Gao et al. [54]	2022	Raw EEG	Dilated CNN	93.3	-	-	0.007
Zhang et al. [55]	2022	EEG Spectrogram	ViT	59.2–97.0	65.8–94.6	-	-
Proposed Method	2022	EEG Scalogram	MViT	99.8	99.7	99.8	0.004

**Table 2:** Benchmarking of the previous seizure-prediction methods and our MViT approach: Kaggle/AES Seizure Prediction dataset.

Authors/ Team	Year	EEG Features	Classifier	SENS (%)	AUC Score Public/Private
Medrr [32]	2016	N/A	N/A	-	0.903/0.840
QMSDP [32]	2016	Correlation, Hurst exponent,	LassoGLM,	-	0.859/0.820
		fractal dimensions,	Bagged SVM,		
		Spectral entropy	Random Forest		
Birchwood [32]	2016	Covariance, spectral power	SVM	-	0.839/0.801
ESAI CEU-UCH [32]	2016	Spectral power,	Neural Network,	-	0.825/0.793
		correlation, PCA	kNN		
Michael Hills [32]	2016	Spectral power, correlation,	SVM	-	0.862/0.793
		spectral entropy, fractal dimensions			
Truong et al. [24]	2018	EEG Spectrogram	CNN	75.0	-
Eberlein et al. [56]	2018	Multi-channel time series	CNN	-	0.843/-
Ma et al. [57]	2018	Spectral power, correlation	LSTM	-	0.894/-
Korshunova et al. [58]	2018	Spectral power	CNN	-	0.780/0.760
Liu et al. [27]	2019	PCA, spectral power	Multi-view CNN	-	0.837/0.842
Qi et al. [28]	2019	Spectral power, variance, correlation	Multi-scale CNN	-	0.829/0.774
Chen et al. [59]	2021	EEG Spectrogram	CNN	82.00	0.746/-
Hussein et al. [29]	2021	EEG Scalogram	SDCN	88.45	0.928/0.856
Usman et al. [60]	2021	statistical and spectral moments	Ensemble of SVM,	94.20	-
			CNN, and LSTM		
Zhao et al. [61]	2022	Raw EEG	CNN	91.77–93.48	0.953–0.977/-
Proposed Method	2022	EEG Scalogram	MViT	90.28	0.940/0.885

## Data Availability

The datasets generated during the current study are available from the corresponding author upon reasonable request.

## Abbreviations

The following abbreviations are used in this manuscript:

AES	American Epilepsy Society
AUC	Area under the ROC Curve
CHB	Children’s Hospital Boston
CNN	Convolutional Neural Networks
CWT	Continuous Wavelet Transform
DCAE	Deep Convolutional AutoEncoder
DFT	Directed Transfer Function
EEG	Electroencephalogram
FPR	False Positive Rate
FFT	Fast Fourier Transform
GCN	Graph Convolutional Network
iEEG	intracranial EEG
LassoGLM	Lasso regularization of Generalized Linear Models
LN	Layer normalization
LSTM	Long Short-Term Memory
MLP	Multi-Layer Perceptron
MViT	Multi-Channel Vision Transformer
MSA	Multi-head Self Attention
NLP	Natural Language Processing
PCA	Principal Component Analysis
Proj	Projection
RNN	Recurrent Neural Networks
ROC	Receiver Operating Characteristic
SAS	Seizure Advisory System
SENS	Sensitivity
STFT	Short-Time Fourier Transform
SPEC	Specificity
SVM	Support Vector Machine
ViT	Vision Transformer
